# LSTM-assisted optical fiber interferometric sensing: breaking the limitation of free spectral range

**DOI:** 10.1038/s41377-025-02008-4

**Published:** 2025-12-01

**Authors:** Junling Hu, Sa Zhang, Meiyu Cai, Mingjian Ma, Shuguang Li, Hailiang Chen, Sigang Yang

**Affiliations:** 1 State Key Laboratory of Metastable Materials Science & Technology and Key Laboratory for Microstructural Material Physics of Hebei Province, School of Science, Yanshan University, Qinhuangdao, 066004 China; 2 Hebei Key Laboratory of Advanced Laser Technology and Equipment, Tianjin, 300401 China; 3 Tsinghua National Laboratory for Information Science and Technology (TNList), Department of Electronic Engineering, Tsinghua University, Beijing, 100084 China

**Keywords:** Fibre optics and optical communications, Optical sensors

## Abstract

Optical fiber interferometric sensors are of great importance in chemistry, biology, and medicine disciplines owing to high-sensitivity and high-quality factor. However, due to the limitation of free spectral range, the inherent trade-off between wide measurement range and high sensitivity poses a persistent challenge in interference sensor development, which has fundamentally hindered their widespread adoption in precision measurement applications. In this work, a long short-term memory neural network is utilized in a Mach-Zehnder interference-based refractive index sensor to break the free spectral range limitation. Unique gating mechanism in long short-term memory neural network enables it to efficiently process long-term dependent sequence information, such as interference spectrum, avoiding the need for complex spectral signal analysis. A one-to-one mapping relationship is established between the interference spectrum and refractive index with root mean square error of 3.029 × 10^−4^ and a coefficient of determination of 0.99971. The measurement range is extended from a single free spectral range of 1.3333–1.3561 to approximately three free spectral ranges of 1.3333–1.3921 without sacrificing sensitivity. Moreover, a wider measurement range can be achieved with sufficient training data. This work successfully resolves the inherent contradiction between high sensitivity and wide dynamic measurement range in optical interference-based sensors, opening up a path for the next generation of intelligent sensing systems.

## Introduction

Optical fiber interferometric sensors are critical for high-precision detection of biological and chemical substances in liquid environments, combining unmatched advantages of real-time response, ultrahigh sensitivity, and anti-electromagnetic interference^1–7^. A prominent application is the rapid determination of glucose levels via refractive index (*RI*) measurements in blood samples for the management of diabetes^8,9^. Nevertheless, these sensors face a fundamental limitation: the free spectral range (FSR) imposes an intrinsic constraint on the detection range^10,11^. Conventional signal analysis methods rely on manually tracking the wavelength shift of interference dips within a fixed spectral window, where sensitivity is quantified by spectral displacements. Unfortunately, high sensitivity often causes the spectral response to exceed an FSR, leading to spectral overlap. While reducing the measurement range can mitigate this problem, such a trade-off severely hinders practical applications.

In 2016, William Morrish et al. fabricated a flow-through optical sensor. Interference images are obtained by having light enter a microcapillary with a mirror channel, and the *RI* sensitivity can reach 1540 nm/RIU^12^. In 2018, Giulia Rigamonti et al. introduced a micro-light transmission sensing platform using rectangular miniature hollow glass tubes, with a *RI* sensitivity of up to 507.5 nm/RIU^13^. In 2024, Zhang et al. demonstrated a cascaded double-taper structure exploiting the Vernier effect, achieving an impressive sensitivity of 9066 nm/RIU in liquid *RI* sensing^14^. Yet, the measurable *RI* range remained confined to just 1.3359–1.3477 due to FSR limitations. To address spectral overlap, a method of integrating a fiber Bragg grating (FBG) into Sagnac interferometer was reported^15^, utilizing FBG’s linear temperature response for spectral labeling. However, FBG-based methods suffer from low sensitivity, difficulty in cleaning. In 2025, Cai et al. attempted to overcome FSR constraints by tracking FSR variations with temperature^16^, but the requirement for manual calculation for each FSR individually compromises practicality. Given these challenges, a robust and cost-effective strategy is urgently needed to extend the dynamic range of optical fiber interferometric sensing without sacrificing sensitivity.

Machine learning has emerged as a powerful tool in fields ranging from medical diagnostics to autonomous systems and pattern recognition^17–23^. Deep learning, a subfield of machine learning^24^, has been introduced to optical fiber interferometric sensing to enhance the device performance. The long short-term memory (LSTM) neural network, a deep learning-based algorithm^25^, has shown great potential in optical fiber interferometric sensing applications. For instance, Jiang et al. leveraged LSTM to demodulate FBG sensor signals by directly extracting Bragg wavelengths from spectrum features, achieving rapid and precise wavelength detection^26^. Guo et al. further validated the exceptional precision of LSTM model in retrieving temperature measurements from FBG sensors^27^. In fact, the spectrum represents sequential intensity distribution as a function of wavelength, typically comprising thousands of data points. Traditional recurrent neural networks (RNN) have difficulty in modeling long-distance dependencies when dealing with data of such long sequences. As a special variant of RNN, LSTM is a sequence-based network model with good context-aware ability. It can associate previous information with the current task and effectively capture the subtle but crucial changes in the sequence information^28,29^, making it significantly advantageous for analyzing sequential data such as transmission spectrum.

Optical fiber interferometric sensing faces a trade-off between sensitivity and measurement range due to the limitation imposed by the FSR. To overcome this limitation, we introduce a well-trained LSTM neural network capable of comprehensively learning and extracting multi-dimensional features from entire interference spectrum. The learned features are directly mapped one-to-one to the target measurands, realizing precise identification of different quantities to be measured. As a proof of concept, we demonstrate wide-range *RI* detection beyond a single FSR using a Mach-Zehnder interference (MZI) sensor manufactured by tapered single-mode fiber (SMF) in this work. Unlike conventional dip tracking method, the proposed novel method effectively overcomes the FSR limitation, extending the *RI* detection range from a single FSR to three FSRs. Furthermore, we evaluate the LSTM model’s demodulation accuracy under varying spectral conditions, including different sampling points and wavelength ranges. The proposed LSTM-assisted interferometer opens new possibilities for high-sensitivity, wide-dynamic-range optical fiber interferometric sensing beyond FSR limitations.

## Results

### Experimental setup

**Fig. 1:**
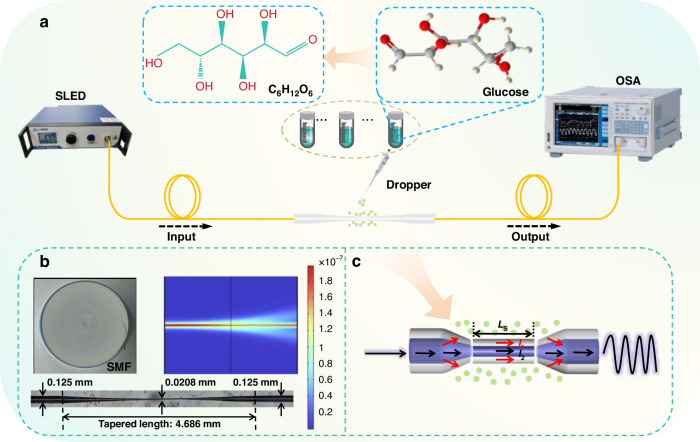
Schematic of a tapered SMF-based MZI sensor for *RI* measurement. **a** Experimental setup for *RI* measurement. **b** Simulated optical path of sensing, along with a microscopic image of a 4.686 mm tapered SMF. **c** Detailed schematic of inner sensing optical path

Figure 1a illustrates the *RI* sensing system based on the MZI structure, which primarily consists of a broadband light source (BBS, Shanghai Aoshow Information Technology Company Ltd., Shanghai, China, SLED), a tapered SMF, a flame-based tapering machine (Guang Xun Tong Technology Co., Ltd, GXT6012) and optical spectrum analyzer (OSA, YOKOGAWA, Tokyo, Japan, AQ6370D). The SLED continuously launches broadband light into the sensor, and an OSA with a resolution of 0.02 nm is used to receive the interference spectrum. The tapered SMF is fabricated using a flame-based tapering machine. After the protective coating is removed, the SMF is fixed on the fixture platform by magnets. The left side of the fiber is connected to the SLED, while the right side captures spectral information in real time by interfacing with the OSA. During this process, the burner and the fixture move according to the preset parameters. When the output spectrum reaches the desired FSR, the machine stops running and the tapered SMF fabrication process is completed. The tapered SMF significantly enhances the leakage ratio of light from the fiber core to the cladding, allowing it to come into direct contact with the liquid to be tested and thereby enabling a rapid response to *RI* changes. Figure 1b presents both the simulated optical field distribution of the tapered SMF sensing region and a microscopic image of the fabricated tapered SMF, which features a tapered region length of 4.686 mm and a central taper diameter of 0.0208 mm. A schematic diagram of the tapered region is schematically illustrated in Fig. 1c. When light passes through the first taper region, it splits into two beams of light. One beam passes through the core for transmission and is labeled as *I*_2_, while the other leaks into the cladding for transmission and is labeled as *I*_1_. When both beams reach the second taper region, they are coupled to the core output, and the resulting light intensity is represented as *I*^30^. The two beams have a constant phase difference and optical path difference, which result in periodic interference spectral lines observed on the OSA.

Glucose solutions of different concentrations were prepared at an ultra-small interval of 0.002 as *RI* matching solutions. During the configuration process, a pipette was used to ensure a constant dosage, and an Abbe refractometer (Beijing Linghang Lijia Electromechanical Company Ltd., MSDR P1-102) was employed for calibration. The Abbe refractometer we use has a built-in temperature compensation function. The values measured for the test liquids are all based on the condition of 20 °C. Additionally, the storage environment for the *RI* matching solution is also 20 °C. Before testing each *RI* matching solution, the values will be calibrated again using the Abbe refractometer. The *RI* of a substance is a function of wavelength and varies with wavelength. To distinguish different substances, physics provides the *RI* values of all substances at the sodium yellow light wavelength, that is, the standard detection wavelength of *RI* is 589.3 nm. According to the *RI* value at this wavelength, the differences or changes between substances can be indicated. Upon full contact between the glucose solution and the sensor, the transmission spectrum of the MZI shifted in response to the external *RI* change. After each measurement, the sensor underwent a rigorous cleaning protocol involving multiple rinses with deionized water until the transmission spectrum returned to its initial state. This cleaning procedure ensured complete removal of residual analytes and restored the sensor’s surface properties for subsequent measurements. The biological structure and chemical formula of glucose molecule are shown in Fig. 1a.

### Challenge analysis

The optical fiber interference sensor is mainly recorded and analyzed by manual tracking of dip wavelength. Tapered MZI has higher sensing sensitivity among various manufacturing methods. However, the high sensitivity may cause the dip wavelength to shift beyond an FSR in a wide range of *RI* detection, leading to spectral overlap, where multiple sensing values correspond to the same wavelength.

**Fig. 2:**
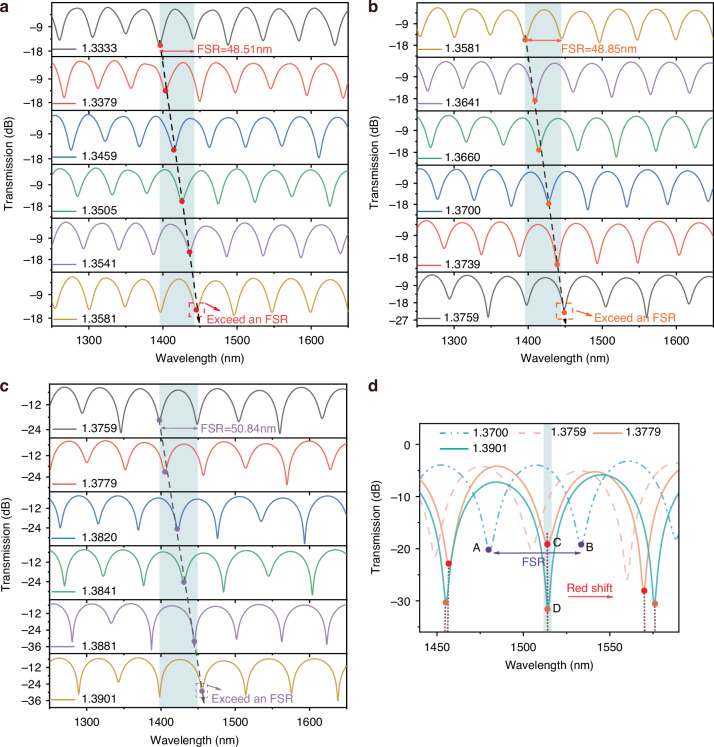
Analysis of FSR Impact on *RI* measurement. The *RI* transmission spectrum based on MZI exceeding FSR of **a** 1.3333–1.3581, **b** 1.3581–1.3759 and **c** 1.3759–1.3901. **d** Diagram of overlapping dip wavelength

As shown in Fig. 2d, the blue dashed line represents the transmission spectrum at an *RI* of 1.3700. Dips A and B define a sensing interval, with A as the initial dip and B as the terminal dip. The sensing interval is marked as an FSR. A redshift in the transmission spectrum is observed with increasing *RI*. Dip A shifts to the spectral position of Dip C at an *RI* of 1.3779. Further increasing the *RI* to 1.3901 causes Dip A to move beyond the distinguishable range, while a new dip (Dip D) emerges between Dips A and B, coinciding spectrally with Dip C at the same wavelength. Crucially, Dips C and D correspond to different *RI* values, making it challenging to determine measured *RI* using conventional dip tracking method. This phenomenon was verified across the entire *RI* measurement range. Figure 2a shows the spectral drift in the *RI* range of 1.3333–1.3581. An interference Dip at 1400 nm is selected for monitoring, corresponding to an FSR of 48.51 nm. The results indicate that the interference dip shift exceeds an FSR. An analogous trend is observed in the *RI* range of 1.3581–1.3901, as shown in Fig. 2b, c. These results highlight a fundamental constraint of conventional demodulation methods, which are inherently limited to tracking spectral shifts within a single FSR. *RI* changes exceeding multiple FSRs introduce ambiguity when demodulating the spectral response. Therefore, developing a simple and efficient strategy to overcome the FSR limitation is crucial for rapid and accurate *RI* recognition over a wide range.

### Configuration of LSTM model

**Fig. 3:**
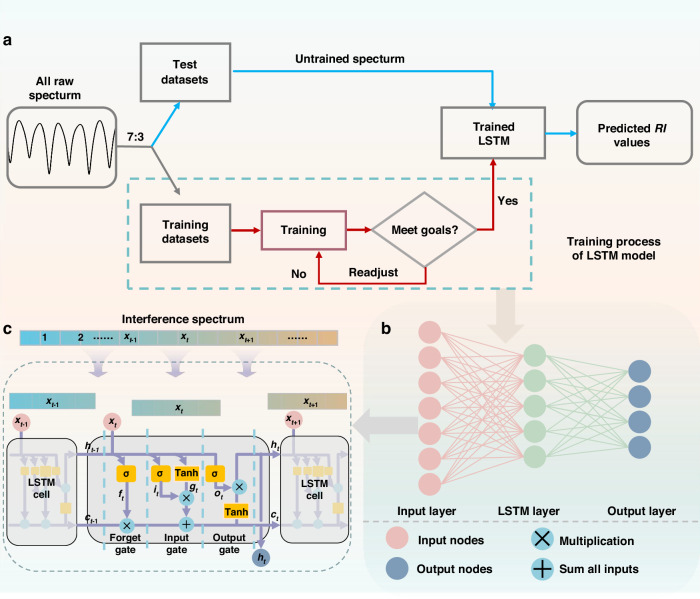
Architecture of spectrum data processing based on LSTM. **a** Flowchart of the proposed LSTM for recognizing spectrum, **b** Schematic structure of LSTM model. **c** Three gate layers of LSTM cell

LSTM has been demonstrated to be suitable for wavelength detection, spectrum demodulation, sequence prediction and other related fields^31–33^. Here, we construct an LSTM model to demodulate spectrum information in interferometric sensing to achieve *RI* recognition. The entire recognition process mainly consists of two steps, as shown in Fig. 3a. The first step involves spectrum data acquisition prior to deep learning. Sufficient interference spectrum data needs to be input into the LSTM model to ensure accurate learning of the internal information. The *RI* of the glucose solutions was incrementally increased from 1.3333 to 1.3939 in steps of 0.002, with 10 transmission spectra recorded at each *RI*, resulting in a total of 320 spectra. The spectrometer used for data collection operates within a wavelength range of 1250–1650 nm, with a wavelength sampling interval of 0.2 nm, so each spectrum contains 2001 sampling points. These spectra datasets serve as training and test data for the subsequent deep learning process. In the second step, a deep learning algorithm is applied to identify the *RI* of an unknown transmission spectrum.

Before training the LSTM model, 70% of the spectra data are randomly selected as the training set and sent into the model. The intensity values of each wavelength sampling point in the transmission spectrum are taken as the learning objects, and each of these sampling points carries its own information. The *RI* corresponding to the transmission spectrum is taken as the learning target. During the training process, the LSTM model utilizes a unique gating mechanism to automatically learn the feature information related to the model’s output variable *RI* from the entire spectral sequence of the sensor, even if the feature information is embedded in the spectral signal. Repeated training iterations are necessary, which can enable LSTM to gradually master which local spectral changes are closely related to *RI*, even if these changes are very subtle. The learned feature information will assist in achieving a one-to-one mapping between the original measurement spectrum and the *RI*, thereby overcoming the limitation of the FSR. After the model training is completed, the remaining 30% of the spectral data is used to test the performance of the model. For a well-trained model with satisfactory performance, the predicted *RI* values should be distributed along the diagonal line *y* = *x* with the actual values.

Figure 3b shows a schematic diagram of the constructed LSTM model. LSTM model captures the internal information of the transmission spectra, such as spectral shape, valley distance and spectral intensity. Learned information is linked to the output (*RI*) through the LSTM, forming a complex regression relationship. It is worth noting that although the conventional RNN model is capable of handling sequences of arbitrary length, it encounters challenges when dealing with long sequences. The repetitive module of RNN contains only a single neural network layer, which easily leads to issues of gradient explosion or vanishing. Unlike RNN, LSTM embeds a storage unit in the hidden layer, as shown in Fig. 3c. The storage unit contains three gates: the input gate, the output gate and the forget gate. Specifically, the input gate determines which new information is stored in the cell state, while the output gate controls which information is transmitted based on the current state. The forget gate provides a mechanism for partially resetting the memory by discarding certain information from the cell state. Through the synergistic effect of these three gates, the propagation of feature information associated with the model output values learned from different spectral bands in the storage unit can be selectively retained or controlled.

Here, intensity of spectral wavelength sampling points at sequence *t* is the input *x*_*t*_ and the corresponding output is *h*_*t*_. *c*_*t*_ is the cell state, which selects and discards information from the current input and cell memory through the Sigmoid function and tanh function. The first step in the LSTM network is to pass the forget gate *f*_t_, which determines which information the cell should discard. *f*_t_ obtains the value from [0,1]. A value of 0 indicates that the information has been completely forgotten, while a value of 1 indicates that the information has been completely stored. The output of forget gate *f*_t_ can be expressed as:

1 $${f}_{t}=\sigma ({W}_{f}{x}_{t}+{U}_{f}{h}_{t-1}+{b}_{f})$$

where *σ* is the activation function, *W*_*f*_, *U*_*f*_, and *b*_*f*_ are weight matrices and a bias associated with the forget gate, respectively.

The second step is that after forgetting some information, the input gate *i*_*t*_ decides which information will to added to the memory unit for update. *g*_*t*_ updates the cell state through the tanh function, which the input *x*_*t*_ and *h*_*t−1*_ can obtain. *i*_*t*_ and *g*_*t*_ can be expressed by:

2 $${i}_{t}=\sigma ({W}_{i}{x}_{t}+{U}_{i}{h}_{t-1}+{b}_{i})$$

3 $${g}_{t}=\,\tanh ({W}_{g}{x}_{t}+{U}_{g}{h}_{t-1}+{b}_{g})$$

where *W*_*i*_, *U*_*i*_, *W*_*g*_, *U*_*g*_, and *b*_*i*_, *b*_*g*_ are the weight matrices and biases of the corresponding parts respectively.

After determining the retained and discarded information, the cell state *c*_*t*_ is updated by combining the outputs of the forget gate *f*_t_ and input gate *i*_*t*_:

4 $${c}_{t}={f}_{t}\ast {c}_{t-1}+{i}_{t}\ast {g}_{t}$$

where * represents element-wise multiplication, *f*_t_ *c_*t*_ defines which information stored in *c*_*t−*1_ is to be forgotten, and *i*_*t*_ *g_*t*_ defines which new information will be added to the cell state *c*_*t*_.

Finally, the output *h*_*t*_ is generated based on the memory block state and the output gate *o*_*t*_ at sequence *t*.

5 $${o}_{t}=\sigma ({W}_{o}{x}_{t}+{U}_{o}{h}_{t-1}+{b}_{o})$$

6 $${h}_{t}={o}_{t}\ast \,\tanh ({c}_{t})$$

where *W*_*o*_, *U*_*o*_ are the weight matrix and *b*_*o*_ is bias defined for the input gate. *c*_*t*_ is the memory block state at current sequence step.

### Network training

**Fig. 4:**
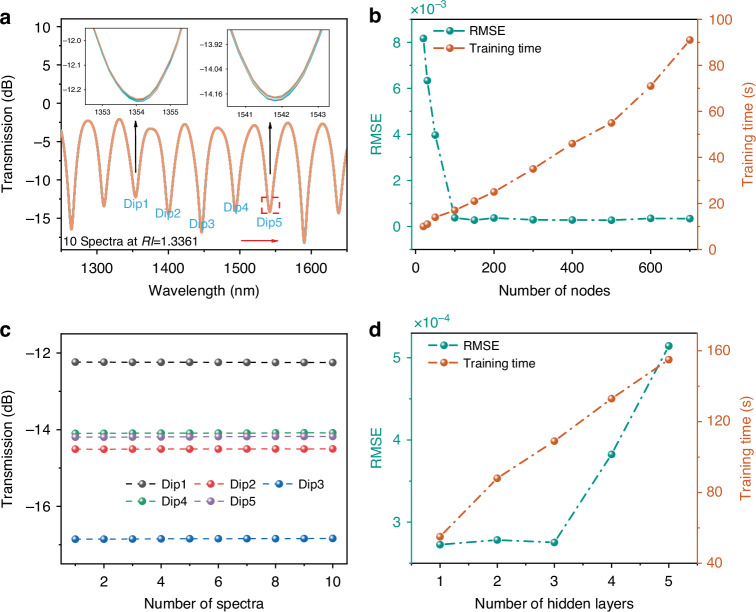
LSTM model configuration for *RI* detection. **a** 10 spectra at an *RI* of 1.3361, insets show the zoomed-in view of two interference dips. **b** *RMSE* and training time of LSTM model of different numbers of hidden nodes. **c** Stability analysis of 10 spectra of Dip1 and Dip5 with *RI* of 1.3361. **d** *RMSE* and training time of LSTM model of different numbers of hidden layers

Figure 4a plots 10 transmission spectra measured at different times for an *RI* of 1.3361, with the inset showing the transmission intensity variations. Figure 4c further illustrates the intensity fluctuations of five selected sensing dips across these 10 spectral measurements. The maximum fluctuation ranges of Dip1–Dip5 are 0.0107, 0.008, 0.02, 0.015 and 0.018 nm, respectively. This disturbance may arise from minor pressure fluctuations resulting from the measurement uncertainties of the hardware itself. As demonstrated by the subsequent prediction results, the LSTM model successfully identifies multiple spectra exhibiting intensity variations under the same *RI*, demonstrating its robustness in demodulating spectral information despite external perturbations. At the same time, we also calculate the relative error values of each Dip at this *RI*, thereby demonstrating the accuracy and precision of this sensor. To make the expression more concise, we average the relative errors of Dip1–Dip5 to obtain values of 0.024098%, 0.015168%, 0.033239%, 0.028111%, and 0.047233%.

In the LSTM model, the 2001 wavelength sampling points of the interference spectrum constitute the input layer, while the corresponding *RI* value constitutes the output layer. Prediction accuracy is a critical aspect of model performance, influenced primarily by two factors: the number of hidden nodes and hidden layers. The Adam optimization algorithm is employed for model training due to its high computational efficiency, low memory footprint, and self-adaptive adjustment of learning rate. All hidden layers use the Rectified Linear Unit (ReLU) activation function, and Root Mean Square Error (*RMSE*) is adopted as the loss function for model optimization. The maximum number of epochs and initial learning rate are set to 1000 and 0.001, respectively.

We first evaluate the influence of hidden node quantity on model performance. LSTM model is trained with different numbers of hidden nodes to obtain the optimal configuration. Figure 4b illustrates the relationship between different numbers of hidden nodes and both *RMSE* and training duration. The results demonstrate that *RMSE* decreases rapidly with increasing nodes before stabilizing, achieving optimal performance at 500 nodes. Meanwhile, training time scales nearly linearly with the number of hidden nodes. Although more hidden nodes enhance the generalization performance of the model, they also increase computational complexity. Here, we choose 500 hidden nodes as the optimal trade-off between precision and efficiency. Next, we study the effect of different hidden layers on model training when the number of hidden units is fixed at 500. All tests are conducted on the same data samples to ensure the reliability of the results. Figure 4d shows the variation of *RMSE* and training time with different hidden layer configurations. The change in the number of hidden layers affects the detection accuracy and training efficiency. The results indicate that *RMSE* initially increases slightly and then rises sharply as the number of layers increases. To maintain accuracy while minimizing computational cost, we choose layers= 1 as the optimal configuration.

### Experimental results and analysis

**Fig. 5:**
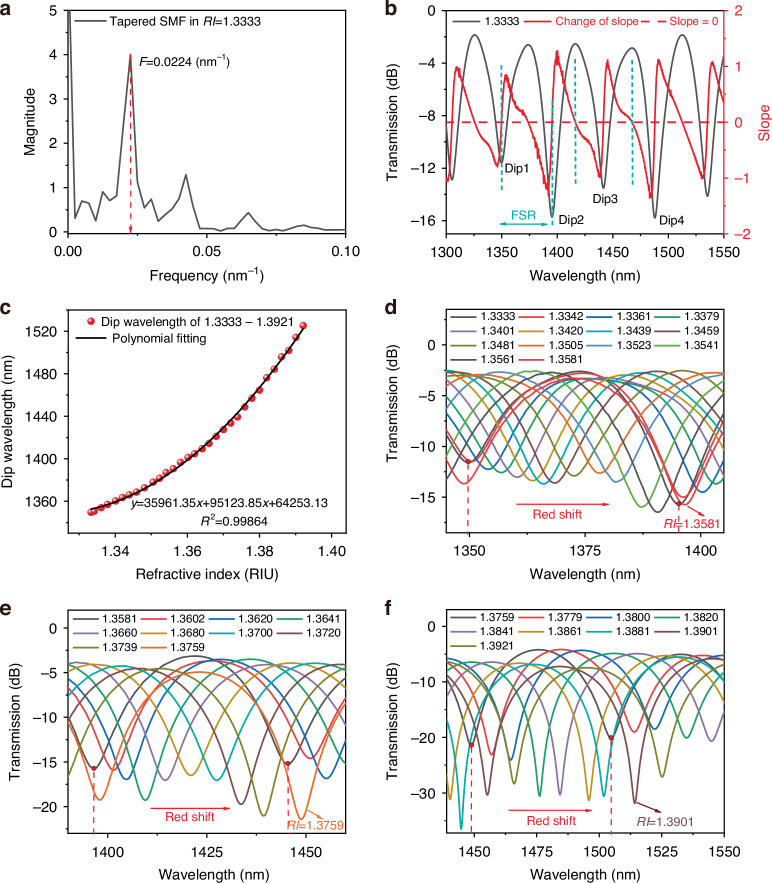
Transmission spectrum characteristics of MZI sensor. **a** FFT of transmission spectrum. **b** The transmission spectrum at an *RI* of 1.3333 (black curve) and the corresponding phase relationship between the two beams (red surve). **c** Nonlinear fitting between *RI* and sensing valley position. **d** Transmission spectrum red shift at an *RI* of 1.3333–1.3581. **e** Transmission spectrum red shift at an *RI* of 1.3581–1.3759. **f** Transmission spectrum red shift at an *RI* of 1.3759–1.3921

The transmission spectrum corresponding to an *RI* of 1.3333 is shown in Fig. 5b, where four adjacent interference dips are selected for analysis. These dips are labeled as Dip1, Dip2, Dip3, and Dip4, respectively. The transmission spectrum undergoes a Fast Fourier Transform (FFT), in which frequency corresponds to the reciprocal of the FSR.

It can be seen from Fig. 5a that the interference phenomenon primarily results from the coupling between the core mode and the cladding mode. This interference phenomenon is governed by the phase relationship between the two beams. When the phase difference satisfies the condition *ϕ* = (2 *m* + 1) π, where *m* = 1, 2, 3, the output is an interference spectrum. In this case, the m-order interference interference dip can be expressed as^34^:

7 $${\lambda }_{dip}=\frac{2\Delta {n}_{eff}{L}_{s}}{2m+1}$$

where *λ* is the wavelength of the incident light in vacuum, *L*_S_ represents the sensing region length, and Δ*n*_eff_ corresponds to the effective *RI* difference between core and cladding modes.

The resolution (*R*) of the sensor is described as:

8 $$R=\frac{\Delta {\lambda }_{\min }}{S}$$

The slope of the transmission spectrum is intrinsically linked to the phase difference between the two interference beams. As shown in Fig. 5b (red lines), while the phase of one beam remains constant, corresponding to the phase change of the other beam. The positive or negative slope directly reflects the direction of phase difference variation. Beyond conventional detection markers such as intensity peaks and dips, feature points in the phase diagram can also serve this purpose. The proposed MZI interferometer leverages phase-modulated sensing, achieving exceptional sensitivity. However, the FSR constrains the monitoring range. Spectral overlap occurs when the spectral shift exceeds an FSR, making it impossible to accurately demodulate the spectral signal. Since the bandwidth of BBS is between 1250–1650 nm, we choose Dip1 for full tracking. Figure 5d–f shows the results of manual tracking spectrum in the *RI* range of 1.3333–1.3921. As shown in Fig. 5d, Dip1 is located at 1349.59 nm at an *RI* of 1.3333. According to Eq. (7), the transmission spectrum red shifts as the *RI* increases, the transmission spectrum line exceeds the first FSR at an *RI* of 1.3581. Dip1 has redshifted to the vicinity of Dip2, and spectral overlap occurs. As shown in Fig. 5e, when the *RI* increases to 1.3759, the transmission spectrum line exceeds the second FSR, and Dip1 has redshifted to the vicinity of Dip3. As shown in Fig. 5f, the transmission spectrum line exceeds the third FSR at an *RI* of 1.3901, and Dip1 has redshifted to the vicinity of Dip4. Figure 5c shows the nonlinear relationship between the full range *RI* and dip wavelength positions, with an average sensitivity of 2986.05 nm/RIU, the measurement range is extended from a single FSR to approximately three FSRs. According to Eq. (8), the *RI* resolution before applying the LSTM model is calculated to be 0.0588, while the *RI* resolution is 0.0248 after applying LSTM. This corresponds to a 2.371 times enhancement in the sensor’s ability to detect the minimum change in *RI*.

### Spectrum demodulation based on LSTM

**Fig. 6:**
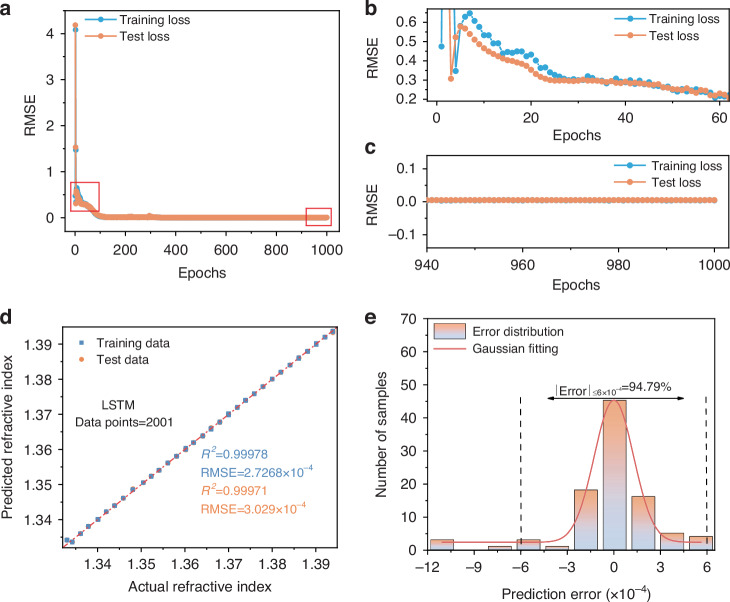
Performance analysis based on LSTM. **a** *RMSE* as a function of the epoch during the training process. **b** The *RMSE* of initial 60 during the training process. **c** The *RMSE* of final 60 epochs during the training process. **d** The predicted *RI* relative to measured situation for the training and test datasets. **e** Histogram of the error between predicted and measured *RI* of the test datasets. The Gaussian fit of the result is represented by the red line

Figure 6a illustrates the relationship between the loss function *RMSE* and epochs of the optimized configured LSTM model. *RMSE* measures the difference between the model’s predicted values and the actual values, with lower *RMSE* values indicating better prediction accuracy. During the first 60 epochs, the model is not adequately trained. Consequently, no better performance is achieved during this period, as shown in Fig. 6b. Poorly trained neural network models tend to produce unreliable and unstable results. It is essential to perform sufficient and long training of the model. As the number of epochs increased, the *RMSE* of both the training and test datasets showed a rapid downward trend. However, a slight increase in the training *RMSE* is observed at around 300 epochs. Therefore, we run the model for 1000 epochs to ensure model convergence. After approximately 1000 epochs, *RMSE* drops to a relatively low level and tends to stabilize, as shown in Fig. 6c, indicating that the model has reached its optimal performance, and is capable of identifying the transmission spectrum with convincing accuracy. The entire training process takes approximately 58 s.

Figure 6d compares measured *RI* with those predicted by the well-trained LSTM model. The predicted values closely match the measured values except for a few data points, demonstrating the model’s high predictive accuracy. The test datasets achieve an *RMSE* as low as 3.029 × 10^−4^ and a coefficient of determination (*R*^2^) exceeding 0.999, further validating the model’s accuracy. To more precisely analyze the error distribution of each test sample, numerical statistics were adopted to quantitatively characterize the prediction errors. The histogram presented in Fig. 6e shows the distribution of errors between predicted and measured *RI* values. The probability of the error smaller than 6 × 10^−4^ is 94.79% and meets the Gaussian distribution, indicating that the majority of predictions have a very small deviation from the true values.

These results demonstrate that the LSTM network can effectively extract critical information directly from raw spectral data, enabling a highly accurate, one-to-one mapping between spectral response and *RI*. More significantly, the integration of LSTM with the optical fiber interferometric sensing system overcomes the limitation imposed by the FSR, extending the effective measurement range from a single FSR to approximately three FSRs. This advancement fundamentally resolves the longstanding trade-off between sensitivity and dynamic range, enabling simultaneous realization of high-sensitivity and wide-range *RI* detection.

### Performance comparison of different LSTM models

The output signal of the optical fiber interference sensor is usually collected in real time through an OSA. Dense wavelength sampling points usually carry more information, thereby generating more accurate results. However, the cost of increasing data sampling points within a specific wavelength range is to extend the scanning time^35^, especially when the measured quantity changes dynamically, high-density sampling may damage the performance of the sensor.

In this section, we discuss the impact on the prediction accuracy of the developed LSTM when the spectral sampling points are significantly reduced. Firstly, a commercial OSA is used to down-sampling the spectra in the wavelength range of 1250–1650 nm in the experiment. The number of sampling points of each spectrum is reduced to 1001 and 401, with the corresponding wavelength intervals increased to 0.4 nm and 1 nm, respectively. Based on the newly collected down-sampling spectra, two new LSTM models are constructed. The original spectral data are directly used as the input of the model, and the corresponding *RI* is used as the output, which maximizes the retention of the characteristics of the original data and also reduces the computational cost during the processing. These models are named DS1001-LSTM model and DS401-LSTM model according to the input dimensions. During the model development, the DS1001-LSTM and DS401-LSTM models adopted the same network architecture and hyperparameter configuration as the previously developed LSTM models. This not only avoids the computational cost caused by re-optimizing the model’s hyperparameters, but also allows for a more reliable evaluation of the model’s performance under different down-sampling conditions. The only difference is in the number of input layer nodes, which reflects different sampling situations^36^.

**Fig. 7:**
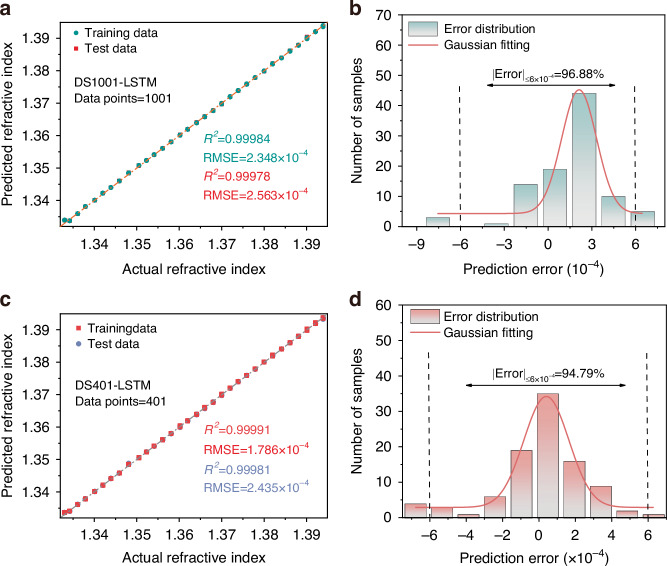
**Performance analysis based on DS-LSTM model**. **a** The predicted *RI* relative to the measured situation for the training and test datasets of DS1001-LSTM. **b** Histogram of the error between predicted and measured *RI* of the test datasets for DS1001-LSTM. The Gaussian fit of the result is represented by the red line. **c** The predicted *RI* relative to the measured situation for the training and test datasets of DS401-LSTM. **d** Histogram of the error between predicted and measured *RI* of the test datasets for DS401-LSTM. The Gaussian fit of the result is represented by the red line

The predictions of the DS1001-LSTM and DS401-LSTM models on both the training and testing datasets are compared with the measured *RI* values, as shown in Fig. 7a, c. Basically, all data points are well distributed along the line *y* = *x*, and the *R*^2^ for *RI* prediction exceeds 0.999 for both down-sampling models. These results indicate that the down-sampling models can effectively predict *RI* with relatively small errors on the training and test datasets. Figure 7b–d further presents the error histograms between the predicted and measured *RI* on the test datasets. For the DS1001-LSTM and DS401-LSTM models, 96.88% and 94.79% of the prediction errors on the test samples remained below 6 × 10^−4^, respectively. Compared with the results of the original LSTM model shown in Fig. 6, the performance of the newly developed DS-LSTM model has not declined.

Although the comparison chart of the actual values and the predicted values can reflect the correlation between the two variables, it cannot quantitatively represent the degree of their correlation^37^. Therefore, Table 1 provides four main model evaluation metrics used to assess the performance of the previously developed LSTM model and two down-sampling models: *MAE*, *RMSE*, Mean Absolute Percentage Error (*MAPE*), and *R*^2^. The ranges of MAE, *RMSE* and *MAPE* are [0, +∞). When the error is large, these values will also be larger. The range of *R*^2^ is [0, 1], with higher value indicating stronger linear correlation between predicted and actual values. In addition, Table 1 also reports the training times of different models. The results show that as the number of sampling points decreases, the *MAE*, *RMSE*, *MAPE* and training time of the down-sampling model decrease, while the *R*^2^ slightly shows a slight increase. This interesting result indicates that the performance of both down-sampling models is superior to that of the original LSTM model, and the prediction accuracy does not decrease despite the significant reduction in spectral sampling points. This can be attributed to the unique gating mechanism of LSTM, which can fully utilize the rich information carried by data points.

**Table 1:** Performance comparison of the test datasets of the three models

Model	*R*^2^	MAE	RMSE	MAPE	Training time
LSTM	0.99971	1.906 × 10^−4^	3.029 × 10^−4^	0.0115%	58 s
DS1001-LSTM	0.99978	1.781 × 10^−4^	2.563 × 10^−4^	0.0131%	37 s
DS401-LSTM	0.99981	1.725 × 10^−4^	2.435 × 10^−4^	0.0127%	25 s

The training times of the DS1001-LSTM and DS401-LSTM models are 37 s and 25 s, respectively, which are significantly lower than that of the original LSTM model. The development of all models is accomplished on standard desktop computers equipped with an Intel Core i5-12400 processor and 32 GB of RAM. It must be noted that down-sampling models still bring certain computational cost, especially during the training stage. However, compared with the original LSTM model, the DS1001-LSTM and DS401-LSTM models, which are based on down-sampling spectra, significantly reduce computational complexity and training time, while simultaneously enhancing prediction performance. Therefore, considering the trade-off between computational burden and model performance, the resulting computational cost introduced by the down-sampling strategy remains within an acceptable range. In conclusion, the developed DS-LSTM models can enhance the system’s update rate by reducing the number of sampling points acquired by the OSA during the measurement process, without compromising measurement accuracy.

## Discussion

A novel LSTM-based demodulation strategy is proposed and successfully demonstrated for *RI* sensing using an MZI fiber sensor, achieving a wide dynamic measurement range beyond the FSR limitation. The results demonstrate that the integration of LSTM with the optical fiber interferometric sensing system significantly enhances demodulation performance, achieving an *RMSE* of 3.029 × 10^−4^ and an *R*^2^ of 0.99971. Notably, the detection range is no longer confined to a single FSR, enabling *RI* detection over a wider range of 1.3333–1.3921. Furthermore, we evaluated the impact of reducing the number of spectral sampling points on demodulation accuracy of the LSTM model. Indeed, the proposed method consistently maintains high prediction accuracy under different sampling point conditions, a challenge for conventional demodulation methods. The integration of LSTM with interference-based optical fiber sensor overcomes the limitation of narrow measurement range imposed by the constraint from the FSR, and realizes precise demodulation of optical fiber interferometric sensing signals faster. A significant advancement in interference-based optical fiber sensors is achieved in this work, paving the way for the development of ultra-low-cost, wide-range and high-sensitivity optical fiber interferometric sensing systems.

## Materials and methods

### Materials

The *RI* sensing system is based on the MZI structure, which primarily consists of a broadband light source (BBS, Shanghai Aoshow Information Technology Company Ltd., Shanghai, China, SLED), a tapered SMF, a flame-based tapering machine (Guang Xun Tong Technology Co., Ltd, GXT6012) and optical spectrum analyzer (OSA, YOKOGAWA, Tokyo, Japan, AQ6370D).

### Device fabrication

The tapered SMF is fabricated using a flame-based tapering machine. After the protective coating is removed, the SMF is fixed on the fixture platform by magnets. The left side of the fiber is connected to the SLED, while the right side captures spectral information in real time by interfacing with the OSA. The burner and the fixture move according to the preset parameters during this process. When the output spectrum reaches the desired FSR, the machine stops running, and the tapered SMF fabrication process is completed. Figure 1b presents a microscopic image of the fabricated tapered SMF, which features a tapered region length of 4.686 mm and a central taper diameter of 0.0208 mm.

## Data Availability

Data will be made available on request.
